# CTRP6(C1q/Tumor Necrosis Factor (TNF)-related protein-6) alleviated the sevoflurane induced injury of mice central nervous system by promoting the expression of p-Akt (phosphorylated Akt)

**DOI:** 10.1080/21655979.2021.1967838

**Published:** 2021-09-13

**Authors:** Zhiwen Liu, Bin Yang

**Affiliations:** Department of Anesthesiology, The Second Hospital, University to South China Hengyang Cty, Hengyang City, Hunan Province, China

**Keywords:** Sevoflurane, central nervous system injury, apoptosis, CTRP6, p-Akt

## Abstract

Postoperative cognitive impairment and nervous system damage caused by anesthetics seriously affect patient’s postoperative recovery. Recent study has revealed that CTRP6 could alleviate apoptosis, inflammation and oxidative stress of nerve cells, thereby relieving nervous system damage induced by cerebral ischemia reperfusion. However, whether CTRP6 could relieve sevoflurane induced central nervous system injury is unclear. We stimulated C57BL/6 mice with sevoflurane and injected CTRP6 overexpression adenovirus vector. Next, H&E staining and TUNEL assays were performed to examine the effect of CTRP6 on sevoflurane induced injury of central nervous system. Finally, we isolated primary nerve cells of hippocampus. Flow cytometry and commercial kits were used for the detection of apoptosis and ROS levels of these cells. Western blotting was used for the detection of the expression level of p-Akt in central nervous tissues and primary cells. Results showed that sevoflurane induced injury and apoptosis of central nervous tissues. Overexpression of CTRP6 relieved apoptosis and injury of these tissues. CTRP6 inhibited the expression of cleaved caspase-3 and cleaved PARP in these tissues. Sevoflurane promoted apoptosis of primary cells and enhanced the expression of ROS and MDA in these cells. Overexpression of CTRP6 alleviated apoptosis and suppressed production of ROS and MDA in these cells. In addition, CTRP6 also enhanced the expression of p-Akt in primary cells. Taken together, our results suggested that CTRP6 relieved sevoflurane induced injury of central nervous tissues by promoting the expression of p-Akt. Therefore, the targeted drug of CTRP6 should be explored for the remission of these symptoms.

## Introduction

Postoperative cognitive dysfunction (POCD) is a common neurological complication after anesthesia surgery, which has become a major issue affecting the recovery process and quality of life of the elderly after surgery [1]. Sevoflurane is a commonly used clinical inhalation anesthetic. And the application of this drug has a greater impact on postoperative cognitive function [2,3]. One study suggested that the using of sevoflurane could induce apoptosis of hippocampal cells of mice [4]. Furthermore, sevoflurane also induced inflammation of cells in hippocampus of mice [5]. However, the mechanism by which this narcotic drug affects cognitive impairment remains unclear. Therefore, in-depth study of the mechanism of sevoflurane-induced postoperative cognitive dysfunction and finding strategies to eliminate the cognitive dysfunction caused by sevoflurane has always been a hot and difficult point in anesthesiology research. In addition, another study showed that central nervous system damage caused by surgery and anesthesia is one of the main reasons for postoperative cognitive dysfunction [6]. The alleviation of central nervous system inflammation could improve postoperative cognitive function and reduce the incidence of POCD [7].

C1q/Tumor Necrosis Factor (TNF)-related Protein-6 (CTRP6) is a member of the adiponectin-like protein family. Although CTRP has structural homology with adiponectin, each CTRP has a unique tissue distribution and exhibits diverse functions [8]. CTRP6 is expressed in adipose tissue, placenta, heart and brain, and has a regulatory effect on energy metabolism and inflammation [9,10]. CTRP6 could mediate the expression of interleukin-10 (IL-10) through the activation of macrophage extracellular signal-regulated kinase 1/2 [11]. Therefore, CTRP has the potential to be a crucial target for the use of drugs to treat inflammatory diseases. At present, some studies have confirmed that CTRP6 could relieve inflammation-related diseases such as myocardial fibrosis [12], heart injury [13] and arthritis [14], but the role of CTRP6 in ischemia-reperfusion injury is rarely reported. Interestingly, as another member of the CTRP family, CTRP9 has been shown to alleviate myocardial ischemia-reperfusion injury by inhibiting apoptosis [15,16]. One study showed that CTRP6 could protect against cerebral ischemia-reperfusion injury by reducing cell inflammation, oxidative stress and apoptosis [17]. However, CTRP6 is rarely studied in central nervous injury caused by anesthesia, and its functional mechanism is still unclear.

In this study, we speculated that the expression of CTRP6 could alleviate the sevoflurane induced injury of central nervous tissues by activating the expression of p-Akt. We examined the efficacy of CTRP6 during the development of the sevoflurane-induced postoperative cognitive dysfunction. And the results of this study could provide potential therapeutic target and strategy for the clinical treatment of POCD patients.

## Material and methods

### Animal assays

Total of 32 male C57BL/6 mice were purchased from the Shanghai Experimental Animal Center of Chinese Academy of Sciences and fed under the Specified Pathogen Free conditions for one week. All these mice were divided into four groups (eight mice per group, Sham group, Sev group, Sev+Ad-NC group and Sev+Ad-CTRP6 group). The mice of Sham group were fed with the normal condition and used as the control group. Mice of Sev group, Sev+Ad-NC group and Sev+Ad-CTRP6 group were kept in a small room equipped with an anesthesia device and a gas monitoring device for 4 hours every day [18]. The mice of Sham group were also kept in the same room without the anesthesia. During the period, mice in Sev group, Sev+Ad-NC group and Sev+Ad-CTRP6 group were stimulated with sevoflurane (Wanshi company, Japan). Sevoflurane (concentration 2.6%) was mixed with the humidified 30% O_2_ carrier gas and was provided by the calibrated vaporizer. The mice were stimulated with the vaporizer for 1 h per day. The mice of Sev+Ad-CTRP6 group and Sev+Ad-NC group were injected with the CTRP6 overexpression adenovirus vector and corresponding negative control. All the adenovirus vectors were designed and obtained from the Genechem (Shanghai, China). After treating the mice with sevoflurane for one week, a water maze experiment was carried out to test the cognitive ability of the mice. Then, these mice were euthanized and their brain tissues were collected for further analysis. The using of these mice followed the animal experimental guidelines which were set up by the National Institutes of Health [19]. This research was approved by the The Second Hospital， University to South China Hengyang Cty.

### Morris water maze task

After stimulating the mice with anesthetics for one week, the water maze experiment was used to study the learning and spatial memory abilities of the mice of these four groups. The mice were placed in the water facing the wall, and then the time of the mice to find an escape platform under the water was recorded. Furthermore, the number of platform crossing was also recorded. The probe trial was performed to detect the memory ability of these mice after 24 hours of spatial acquisition trial. In this part, we removed the platform from the pool and induced the mice to swim in the pool for 2 minutes in any of the four parts of the swimming pool. During the process, the path length of swimming, swimming speed and time spend in the target quadrant was recorded with the computer. The proportion of the swimming time in the platform placed quadrant in the total time is considered to be the crucial indicator of the memory ability of mice.

### Hematoxylin eosin staining (H&E staining)

The brain tissues of these mice were collected after sacrificing these mice. Xylene was used to dewax and dehydrate these tissues. Next, these tissues were stained with the hematoxylin solution (Thermo Fisher Scientific, USA) for 5 minutes. Then, we washed these tissues with the double distilled water. After that, these tissues were washed with the 0.1% HCl solution and stained with the eosin solution (Thermo Fisher Scientific, USA) for 2 minutes. Finally, we washed these tissues with the double distilled water and dehydrated these tissues. These tissues were observed under the microscope (Olympus, Japan).

### Immunofluorescence assay

Mouse brain tissues were deparaffinized and hydrated, and then the antigen was extracted with sodium citrate buffer. Next, these tissues were blocked with goat serum for 1 h at room temperature. Then, these tissues were incubated with the antibodies at 4°C overnight. The antibodies used in this research were NeuN (Abcam, ab177487) and CTRP6 (Abcam, ab36900). In the second day, these tissues were incubated with Alexa Fluor 647 (red for CTRP6, cell signaling technology, USA) and Alexa Fluor 488 (green for NeuN, cell signaling technology, USA) for 2 hours. DAPI (Thermo Fisher Scientific, USA) was used for the staining the cell nuclei. Finally, these tissues were observed with the laser scanning confocal microscope (Olympus, Japan). These images were analyzed with Image J (National Institutes of Health, USA). The proportion of positive cells in Sev group, Sev+Ad-NC group and Sev+Ad-CTRP6 group were normalized to the Sham group.

### Tunel assay

Tunel commercial kit (Roche, Switzerland) was used for the detection of apoptosis of brain tissues of mice. Image J was used for the analysis of the images. DAPI (Thermo Fisher Scientific, USA) was used for staining the cell nuclei. All the procedures in this assay followed the instructions of the manufacturer.

### Culture of primary cells

We isolated the primary neuronal cells of the mouse hippocampus during the sacrifice of the mouse (four weeks old, C57BL/6). Next, we cultured these cells with the RPMI-1640 medium (Hyclone, USA) supplemented with the 10% fetal bovine serum (Gibco, USA). Then, these cells were transfected with the CTRP6 overexpression adenovirus and corresponding negative control. The cells of Sev group, Sev+Ad-NC group and Sev+Ad-CTRP6 group were stimulated with the sevoflurane (2% concentration).

### CCK-8 assay

Primary cells were plated into 96 well plates. After 24 hours, CCK-8 (Dojindo, Japan) was mixed with the culture medium (1:10) and added into the 96 well plates. Then these cells were placed into the incubator for 1 h. Finally, the absorbance of these cells was measured with the spectrophotometer (Thermo Fisher Scientific, USA).

### Apoptosis assay

Primary cells were stimulated with sevoflurane. After that, trypsin (Beyotime, China) was used to make these cells into single cell suspension. These cells were washed with PBS to clear the residual serum. Then, these cells were incubated with the Annexin V and PI (Beyotime, China) for 40 minutes. Finally, apoptosis of these cells was detected with flow cytometry.

### Detection of ROS

Dichlorofluorescein diacetate (DCFH-DA) was used for the detection of ROS in these primary cells. DCFH-DA (Beyotime, S0033) was diluted with the serum-free medium and incubated with these cells for 40 minutes. Then, these cells were washed with double distilled water in the dark room. Finally, ROS in these cells was observed with a laser confocal microscope (Olympus, Japan) [20].

### Detection of MDA and SOD

Commercial kits (Beyotime, China) were obtained for the detection of the levels of MDA and SOD in primary cells. All the procedures in these assays followed the instructions of the manufacturers.

### Western blotting

The protein samples were collected with the RIPA buffer (Beyotime, China). After that, BCA method was used for the detection of the concentration of these protein samples. Then, these protein samples were separated with 10% SDS-PAGE gel (Beyotime, China). Next, these proteins were transferred to the PVDF membranes (Millipore, USA). Then these membranes were blocked with the BSA (Beyotime, China) for 2 hours. Then, these membranes were incubated with the primary antibodies at 4°C overnight. The primary antibodies used in this research were Cleaved caspase-3 (Abcam, ab2302), Cleaved PARP (Abcam, ab32064), p-Akt (Abcam, ab18785), Akt (Abcam, ab64148) and GAPDH (Abcam, ab8245). All these primary antibodies were diluted with the BSA with the ratio (1:1000). On the second day, these cells were washed with the PBST and incubated with the second antibodies (Abcam, ab205718) for 2 hours in room temperature. The secondary antibodies were diluted with the BSA with the ratio (1:2000). Finally, the immunoreactive signals were measured with the Pierce Western Blotting Substrate (Thermo Fisher Scientific, USA). And the results were quantified and analyzed with Image J software (National Institutes of Health, USA) [21].

## Statistical analysis

All the data in this study was analyzed with GraphPad Prism 6.0. All the data in this study was displayed as the mean ± SD. All the experiments were repeated for three times in this research. Student’s t test was used for the comparison between diverse groups. The difference between diverse groups was considered statistically significant when the value of *p* was less than 0.05.

## Results

The application of anesthetic could induce the POCD. Recently, studies revealed that the expression of CTRP6 has the potential to relieve the apoptosis, oxidative stress and inflammation of PC12 cells. In this research, we speculated that the expression of CTRP6 has the potential to alleviate the sevoflurane induced injury of brain tissues. For *in vivo* study, we used the sevoflurane to stimulate mice and established the CTRP6 overexpression mice. Finally, the assays were performed for the detection of cognition and memory of the mice.

### Overexpression of CTRP6 alleviated the sevoflurane induced injury of central nervous tissues

**Figure 1.**
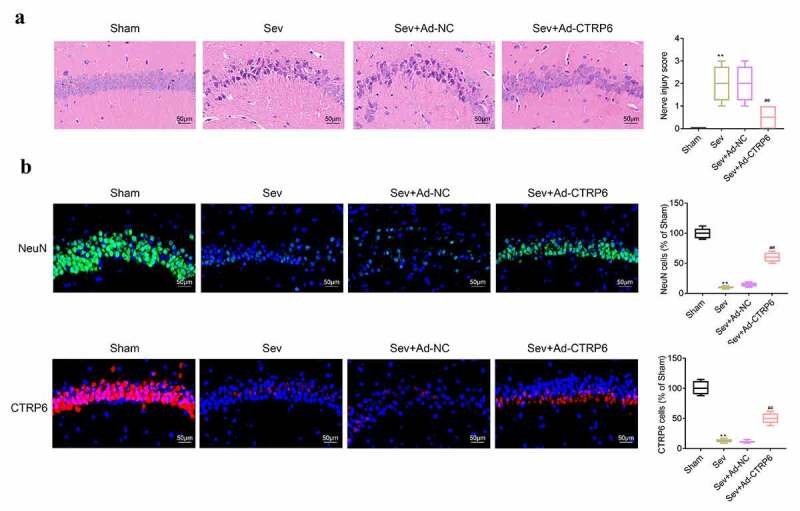
CTRP6 relieved the sevoflurane induced injury of central nervous tissues. (a) H&E staining was performed for the detection of the injury of central nervous tissues. (b) Immunofluorescence assay was performed to detect the expression of CTRP6 and NeuN in these tissues. #*p*< 0.05, **p< 0.01

After mice were sacrificed, H&E staining was performed to detect the injury of the nerve cells in the hippocampus. According to the results (Figure 1a), we found that the number of nerve nucleus was decreased and became deformed after stimulation with sevoflurane. However, the status of nerve cells was normal in the brain tissues of CTRP6 overexpression mice. The expression of CTRP6 and NeuN has protective effects on the injury of central nervous tissue [22]. Therefore, we measured the expression of the CTRP6 and NeuN in brain tissues of these mice by immunofluorescence assay. As shown in Figure 1b, the expressions of CTRP6 and NeuN in these tissues were inhibited after stimulation of sevoflurane. The levels of CTRP6 and NeuN were increased after the overexpression of CTRP6.

### Overexpression of CTRP6 relieved the sevoflurane induced apoptosis of central nervous tissues

**Figure 2.**
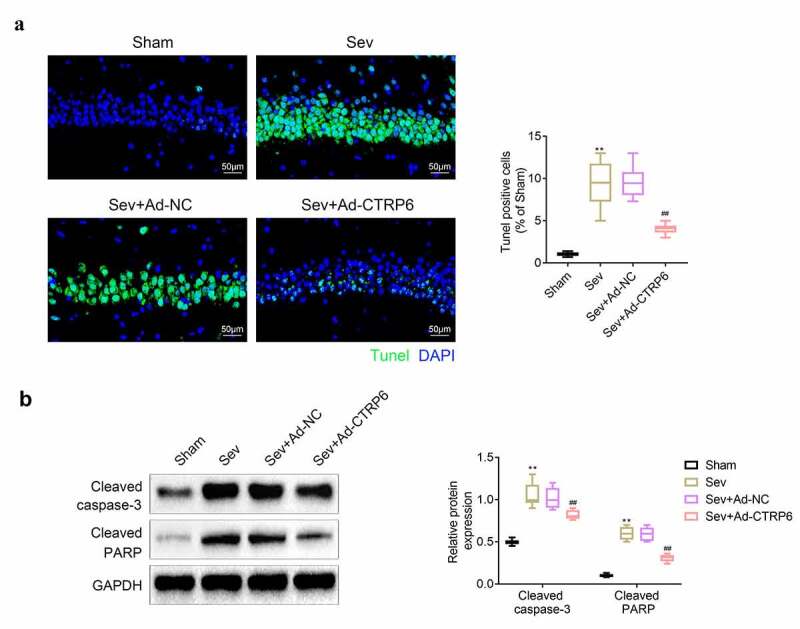
CTRP6 alleviated the sevoflurane induced apoptosis of central nervous tissues. (a) Apoptosis of central nervous tissues was detected with the tunel staining. (b) Western blotting was performed to measure the expression of apoptosis related proteins in central nervous tissues. #*p*< 0.05, **p< 0.01

Apoptosis of nerve cells in the hippocampus could also cause severe cognitive impairment [23]. Thus, TUNEL staining was performed to detect the apoptosis of the brain tissues of these mice. The results (Figure 2a) showed that apoptotic cells in brain tissues were increased after sevoflurane stimulation. The apoptosis in central nervous tissue was inhibited after the overexpression of CTRP6. Next, the expressions of apoptosis related proteins in brain tissues were determined with western blotting. The results (Figure 2b) showed that the expression levels of cleaved caspase-3 and cleaved PARP were enhanced in these tissues after sevoflurane stimulation. However, the levels of these proteins were suppressed after overexpression of CTRP6.

### Overexpression of CTRP6 rescued the memory and learning ability of mice

**Figure 3.**
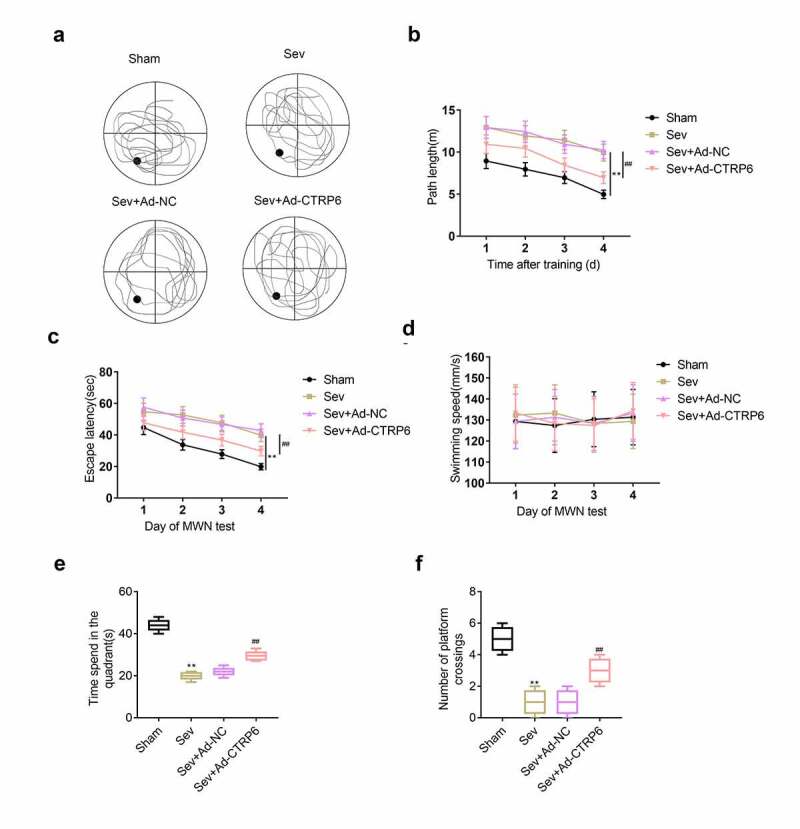
CTRP6 recovered the learning and cognitive abilities of mice. (a) The swimming path of mice were recorded. (b) The length of swimming path was recorded with the computer. (c) The escape latency of these mice in the pool was recorded. (d) Swimming speed of these mice was recorded. (e) Time spend in the platform quadrant of these mice was recorded. (f) Number of platform crossings of these mice was recorded with the computer. #*p*< 0.05, **p< 0.01

In this part, Morris water maze test was performed to explore the memory and learning ability of mice after stimulation with sevoflurane. Results (Figure 3a) showed that the proportion of the movement path of mice in the quadrant without the platform was significantly increased after the stimulation of sevoflurane. However, the proportion of the path in the platform quadrant was rescued after the overexpression of CTRP6. Next, we also found that the length of the movement path and the escape latency were also enhanced after the stimulation of the sevoflurane. And the length of the movement path and escape latency were repressed after overexpression of CTRP6 (Figure 3b and Figure 3c). Finally, we measured the swimming speed, time spend in the platform quadrant and number of platform crossings. As shown in Figure 3d, there was no significance difference of swimming speed between these groups. Interestingly, we found that the time spend in the platform quadrant and number of platform crossings were decreased after the stimulation of sevoflurane. And the values of these indicators were recovered after the overexpression of CTRP6 (Figure 3e and Figure 3f).

### CTRP6 protected primary cells of nerve tissue from sevoflurane induced apoptosis

**Figure 4.**
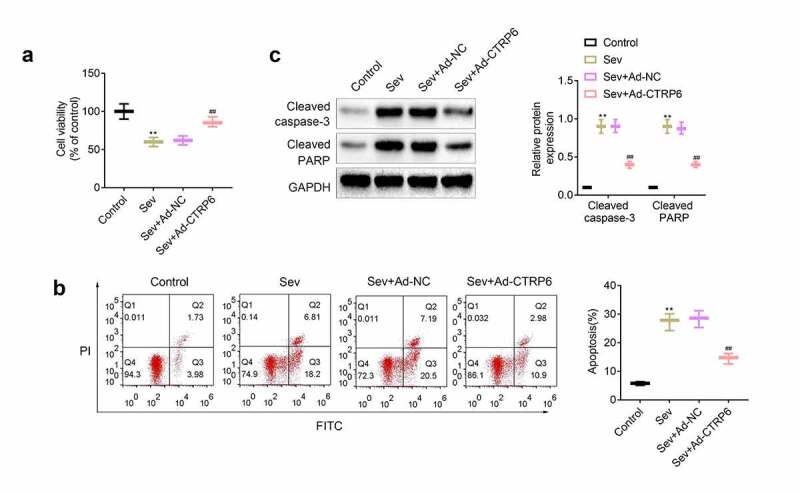
Overexpression of CTRP6 relieved the sevoflurane induced apoptosis of primary cells of central nervous tissue. (a) CCK-8 assays were performed to measure the viability of the primary cells. (b) Flow cytometry was performed to determine the apoptosis of these cells. (c) Western blotting was applied for measuring the expression of apoptosis related proteins in these primary cells. #*p*< 0.05, **p< 0.01

In order to further study the role of CTRP6 in protecting nerve cells from sevoflurane-induced damage, we further isolated and cultured primary cells of neural tissue in the hippocampus of mice. CCK-8 assays were performed to measure viability of these cells in diverse groups. Results (Figure 4a) showed that the viability of primary cells were inhibited after the stimulation of sevoflurane, and the cell viability of these cells were recovered after the overexpression of CTRP6. Next, apoptosis and apoptosis associated proteins were determined with flow cytometry and western blotting. As shown in Figure 4b and Figure 4c, stimulation of sevoflurane promoted the apoptosis of primary cells and the expression of cleaved caspase-3 and cleaved PARP. Furthermore, overexpression of CTRP6 alleviated the apoptosis of these cells and suppressed the expression of cleaved caspase-3 and cleaved PARP.

### CTRP6 relieved the sevoflurane induced oxidative stress of primary cells of nerve tissue

**Figure 5.**
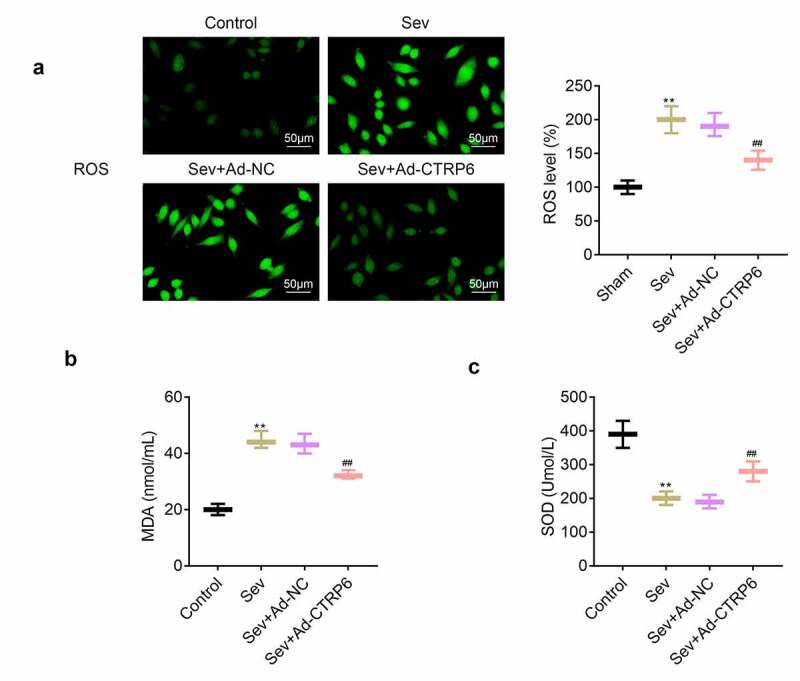
Overexpression of CTRP6 alleviated the sevoflurane induced oxidative stress of primary cells of central nervous tissue. (a) Probe was used for the detection of the ROS levels in these primary cells. (b, c) Commercial kits were used for the measurement of the levels of MDA and SOD in these primary cells. #*p*< 0.05, **p< 0.01

Higher levels of ROS and MDA could also induce oxidative damage of central nervous tissue. In this part, we used the commercial kits to measure the ROS and MDA levels in these primary cells. According to the results (Figure 5a and Figure 5b), we found that the ROS and MDA levels in these cells were increased after the stimulation of sevoflurane. However, the production of ROS and MDA was suppressed in these cells overexpressed CTRP6. In addition, the levels of SOD were decreased after the stimulation of sevoflurane. And overexpression of CTRP6 in these cells rescued the expression of SOD (Figure 5c).

### CTRP6 activated the expression of p-Akt in primary cells of nerve tissue

**Figure 6.**
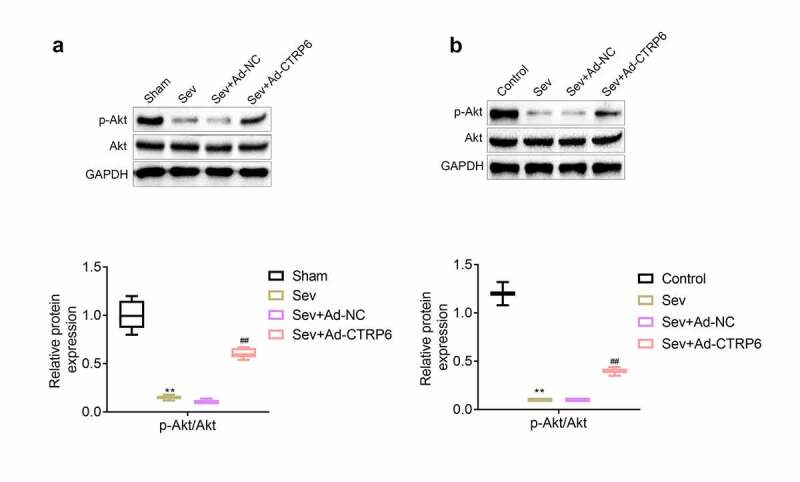
CTRP6 activated the expression of p-Akt in primary cells of central nervous tissue. (a, b) The expression levels of p-Akt and Akt in central nervous tissues and primary cells were measured with western blotting. #*p*< 0.05, **p< 0.01

One study revealed that CTRP6 could relieve the doxorubicin-induced apoptosis of myocardial cells by promoting the expression of p-Akt [13]. Therefore, we determined the expression levels of p-Akt and Akt in nerve tissues of the hippocampus and primary cells by the western blotting. Results showed that the expression of p-Akt in nerve tissues of the hippocampus (Figure 6a) and primary cells (Figure 6b) was decreased after the stimulation of sevoflurane. However, the levels of the protein in the tissue and cells were increased after the overexpression of CTRP6.

## Discussion

The use of anesthetics relieved the pain of many patients during surgery process. However, application of anesthetics also induced cognitive impairment and memory impairment after surgery, which seriously affected patients’ life quality [24]. Sevoflurane is a common anesthetic. One study revealed that the application of sevoflurane induced neuronal oxidative stress and cognitive impairment of rats [25]. In addition, research also revealed that sevoflurane also led to the postoperative cognitive dysfunction of human neuronal cells [26]. Some studies revealed that the occurrence and development of anesthetics induced postoperative cognitive impairment was associated with the inflammation, oxidative stress and apoptosis of cells of central nervous tissues [27,28]. In this study, we also found that stimulation of sevoflurane induced the damage of central nervous tissues. And the application of sevoflurane also induced the apoptosis of central nervous tissue cells of mice. Furthermore, stimulation of sevoflurane also led to the decreased time spend in the platform quadrant and number of platform crossings during the process of water maze experiment. And the stimulation of sevoflurane also aggravated the apoptosis and promoted the production of ROS and MDA in the primary nerve cells of mice hippocampus. These results also indicated that the stimulation of sevoflurane induced the injury of central nervous tissues of mice and led to the damage of the cognitive and memory abilities of mice.

In addition, the proteins of the CTRP family are highly conserved proteins, and these proteins all have a domain similar to adiponectin [29]. CTRP6 is also a protein of this family. One study showed that higher levels of CTRP6 could alleviate the high glucose-induced oxidative stress, inflammation and accumulation of extracellular matrix of mesangial cells [30]. Furthermore, the expression of CTRP6 could also relieve the inflammation of adipose tissue during the occurrence and development of obesity [31]. Recent study also revealed that higher levels of CTRP6 alleviated the cerebral ischemia-reperfusion injury by inhibiting the aggravation of inflammation, apoptosis and oxidative stress of central nervous tissue cells [17]. In this study, we also found that the injury of central nervous tissues was relieved after overexpression of CTRP6 in these mice. Furthermore, the levels of NeuN and the apoptosis cells were decreased after the overexpression of CTRP6 in these mice. Results of water maze experiment also showed that time spend in the platform quadrant and number of platform crossings were recovered after overexpression of CTRP6 in these mice. Meanwhile, overexpression of CTRP6 also relieved the apoptosis and suppressed the production of ROS and MDA in these primary nerve cells. One study suggested that the Urolithin A could alleviate the memory impairment of mice by suppressing the apoptosis of nerve cells [32]. All these results also proved that CTRP6 has the mitigative effect on the sevoflurane induced central nervous tissue damage and cognitive impairment of these mice.

Furthermore, some studies suggested that activation of Akt and p-Akt pathway could relieve the inflammation, apoptosis and oxidative stress in multiple types of cells [33,34]. On the other hand, the increase in p-Akt could also relieve the acrylamide-induced axonal and myelinated damage [35]. Higher level of p-Akt also alleviated the inflammation of microglial cells [36]. In addition, the expression of p-Akt relieved the injury of central nervous tissue induced by cerebral ischemia reperfusion [37]. One study revealed that CTRP6 alleviated the cerebral ischemia reperfusion injury by enhancing the expression of p-Akt [17]. In this study, we also found that the stimulation of sevoflurane induced reduced level of p-Akt in central nervous tissues and primary nerve cells. However, the level of p-Akt was increased after the overexpression of CTRP6.

## Conclusion

Above all, we examined the effects of CTRP6 on the sevoflurane induced injury of central nervous tissue and cognitive disorder in this study. Our results revealed that overexpression of CTRP6 relieved the sevoflurane induced injury of central nervous and recovered the learning and cognitive skills of. The results also suggested that the vital function of CTRP6 in the sevoflurane induced injury of central nervous system.

## Data Availability

All data generated or analyzed during this study are included in this published article.

## Highlight

1. Overexpression of CTRP6 alleviated the sevoflurane induced injury of central nervous tissues

2. Overexpression of CTRP6 relieved the sevoflurane induced apoptosis of central nervous tissues

3. Overexpression of CTRP6 rescued the memory and learning ability of mice

4. CTRP6 protected primary cells of nerve tissue from sevoflurane induced apoptosis

5. CTRP6 relieved the sevoflurane induced oxidative stress of primary cells of nerve tissue

## Abbreviations

POCD: Postoperative cognitive dysfunction

TNF: Tumor Necrosis Factor

CTRP6: C1q/Tumor Necrosis Factor (TNF)-related Protein-6
